# Adherence to Plant-Based Dietary Patterns and Digestive Cancers: A Scoping Review

**DOI:** 10.3390/nu18050756

**Published:** 2026-02-26

**Authors:** Alejandro Oncina-Cánovas, Luis Cabañas-Alite, Iris Comino, Vicente Mustieles

**Affiliations:** 1 Instituto de Investigación Sanitaria y Biomédica de Alicante (ISABIAL), Departamento de Salud Pública, Hª de la Ciencia y Ginecología, Universidad Miguel Hernández, 03010 Alicante, Spain; aoncina@umh.es; 2 Unidad de Epidemiología de la Nutrición, Departamento de Salud Pública, Hª de la Ciencia y Ginecología, Universidad Miguel Hernández (UMH), 03550 Alicante, Spain; 3 CIBER Epidemiología y Salud Pública (CIBERESP), Instituto de Salud Carlos III, 28034 Madrid, Spain; 4 Faculty of Health Sciences, Universidad Europea Miguel de Cervantes, C. del Padre Julio Chevalier 2, 47012 Valladolid, Spain; 5 Public Health Research Group, University of Alicante, 03690 Alicante, Spain; iris.comino@ua.es; 6 Instituto de Investigación Biosanitaria de Granada (ibs GRANADA), 18012 Granada, Spain; vmustieles@ugr.es; 7 Center for Biomedical Research, University of Granada, 18016 Granada, Spain; 8 Consortium for Biomedical Research in Epidemiology and Public Health, 28029 Madrid, Spain; 9 Servicio de Radiodiagnóstico, Hospital Universitario Clínico San Cecilio, 18016 Granada, Spain

**Keywords:** review, dietary patterns, gastrointestinal cancer

## Abstract

**Background/Objectives**: Digestive cancers are among the leading causes of death worldwide. Although their etiology is not fully understood, diet is an important modifiable risk factor. This scoping review aimed to explore the existing evidence on the association between adherence to various plant-based dietary (PBD) patterns and the risk of major digestive cancers. **Methods**: The scoping review followed the Arksey and O’Malley framework and Joanna Briggs Institute recommendations, and results were reported according to PRISMA-ScR guidelines. A systematic search was performed in PubMed/MEDLINE, Scopus, EMBASE, and Web of Science between January 2020 and May 2025. Original observational studies and clinical trials in English or Spanish examining the association between PBD patterns and digestive cancers were included. **Results**: A total of 24 studies were identified, including 16 prospective cohort studies and 8 hospital-based case–control studies, conducted mainly in Europe (*n* = 10), North America (*n* = 8), and Asia (*n* = 6). Most studies used food frequency questionnaires and predefined PBD indices, particularly the plant-based diet index (PDI), healthful (hPDI), and unhealthful (uPDI) (*n* = 13), while others assessed pro-vegetarian (*n* = 2) or EAT-Lancet dietary patterns (*n* = 3). Most studies reported protective associations with all digestive cancer localizations examined, particularly in relation to healthful PBD patterns: colorectal (13/15), pancreatic (6/7), liver (4/4), esophageal (4/5), stomach (3/4) and oropharyngeal (2/2) cancers. On the contrary, unhealthful PBD patterns were linked to a higher risk. **Conclusions**: Overall, the findings of this review highlight that the quality of PBD patterns is crucial for digestive cancer risk. PBD patterns emphasizing whole and minimally processed plant foods were protective, while those characterized by refined or ultra-processed plant products were deleterious. A future standardization of PBD indices would help to improve comparability among studies.

## 1. Introduction

Gastrointestinal cancers are defined as those cancers that mainly affect one or more organs of the digestive system. Among them, the most notable are cancers of the esophagus, stomach, pancreas, liver, and colon. According to data from the Global Cancer Observatory, in 2022, these cancers were among the top 10 deadliest, ranked in the following order: colorectal (2nd), liver (3rd), stomach (5th), pancreas (6th), and esophagus (7th) [1]. Although the etiology of some of them, such as pancreatic cancer, is still not well understood, there are several well-known risk factors, such as socioeconomic status [2] and certain lifestyle habits, including alcohol and tobacco consumption, as well as diet [3]. Notably, diet influences pancreatic cancer risk through multiple metabolic pathways, including insulin resistance, chronic hyperglycemia, obesity-related inflammation, and metabolic dysfunction [4].

Diet is one of the main modifiable risk factors for the prevention of non-communicable chronic diseases, including digestive cancers [5]. Traditionally, research has focused on specific macronutrients or individual foods. However, the study of dietary patterns has become more relevant over time [6], with a special focus on plant-based dietary (PBD) patterns, which have shown the most consistent health benefits [7]. Within these patterns, vegetarian diets are particularly relevant because they represent one of the most extensively studied and well-defined [8]. Vegetarian diets are exclusion-based patterns in which animal-derived foods such as meat, fish, eggs, and dairy are reduced or completely excluded [9]. A recent meta-analysis of observational studies included more than 600,000 participants and found that, compared to non-vegetarian diets, vegetarian diets were associated with a 59% lower risk of stomach cancer and a 15% lower risk of colorectal cancer (CRC), but were not related to other upper gastrointestinal cancers. In addition, these dietary patterns were associated with lower gastrointestinal tumorigenesis in men, but not in women [10].

Although vegetarian diets have also been linked to other health benefits, the prevalence of vegetarians in Western populations remains low [11], which has led to the development of more flexible dietary approaches, such as the so-called pro-vegetarian patterns (PVG) [12]. These PVG patterns focus on prioritizing plant foods rather than excluding animal-based ones. Also, because not all plant foods are equally healthy, healthful (hPVG) and unhealthful (uPVG) variants have been developed [13]. Greater adherence to these PVG patterns has also been associated with a lower risk of esophageal, stomach, and pancreatic cancers, although their role in other digestive cancers remains unknown [14,15]. In this context, new perspectives have also emerged, such as the use of PBDs, whose definition is not as straightforward as the previous ones. PBDs are qualitatively defined as a dietary pattern in which foods of animal origin are totally or mostly excluded, which means that other diets, such as flexitarian diets, could be considered part of this pattern [16].

The human diet not only influences human health but also has a critical impact on the environment and global ecosystems. As such, there is an urgent need to identify and promote dietary patterns that can better support sustainable food systems, simultaneously optimizing human and planetary health. Indeed, PBDs are linked to a consistently lower environmental burden [17], and currently are the best path forward for humanity and the planet as proposed by the recent EAT-Lancet Commission [18].

While PBD patterns could be a promising tool for the prevention of gastrointestinal cancers, there are still knowledge gaps regarding their association with certain subtypes of these cancers. Therefore, we aimed to carry out a scoping review to map the existing scientific evidence by answering the following research question: *What is the existing evidence on the association between adherence to PBD patterns and the risk of digestive cancers (CRC, pancreatic, liver, esophageal and stomach)?*

## 2. Materials and Methods

This review was conducted following the methodological framework established by Arksey and O’Malley [19], and guided by the Joanna Briggs Institute’s recommendations for scoping reviews [20]. The reporting of findings followed the PRISMA-ScR checklist [21]. The review protocol was registered in the Open Science Framework (OSF), available at: https://doi.org/10.17605/OSF.IO/5VQ4K.

### 2.1. Search Strategy

A systematic and comprehensive search was conducted across four databases: Pubmed/MEDLINE, Scopus, EMBASE, and Web of Science (WOS) on 27 May 2025. The search strategy combined controlled vocabulary terms (e.g., MeSH, Emtree) and free-text terms, organized into two thematic blocks: (1) PBD patterns and (2) gastrointestinal cancers or neoplasms.

We applied the following search strategy across all databases, using the same search terms and four different combinations. (1) We focused on PBD patterns: plant-based diet OR plant-based index OR plant-based indices OR PDI OR hPDI OR uPDI OR pro-vegetarian OR provegetarian OR PVG OR hPVG OR uPVG OR EAT-Lancet diet OR mediterranean diet OR vegetarian diet OR planetary health diet; (2) we targeted cancers of the gastrointestinal tract: gastrointestinal cancer OR digestive cancer OR pancreatic cancer OR esophageal cancer OR oesophageal cancer OR stomach cancer OR gastric cancer OR liver cancer OR colorectal cancer; and (3) we combined both sets of exposure and outcome terms using a Boolean AND operator. Finally, (4) we limited the search to studies published within the last 5 years. This time restriction was applied to capture the most recent evidence, given the rapid evolution of research on PBD indices in the last decade. The number of records retrieved from each database was as follows: MEDLINE (*n* = 234 in the last 5 years), Scopus (*n* = 396 in the last 5 years), EMBASE (*n* = 628 in the last 5 years), and WOS (*n* = 515 in the last 5 years). The full search strategies and detailed results for each database are presented in Table 1.

### 2.2. Inclusion and Exclusion Criteria

This scoping review included original research studies published in English or Spanish and available in full text. Eligible studies evaluated the association between PBD patterns and gastrointestinal cancers, specifically pancreatic, esophageal, gastric, liver, or CRC. Designs considered included observational studies (cross-sectional, cohort, case–control) and clinical trials.

Studies were excluded if they were published in languages other than English or Spanish, lacked full-text availability, focused on non-gastrointestinal cancers or non-cancer outcomes, or assessed dietary patterns not centered on plant-based components. Additionally, studies conducted in animals (e.g., rodent models), in vitro experiments, and non-original articles (such as systematic reviews, meta-analyses, editorials, letters, commentaries, and study protocols) were also excluded.

PBD patterns were defined based on the MeSH descriptor “Plant-Based Diets” (MeSH ID: D000095500), which refers to dietary approaches that emphasize the consumption of plant-derived foods (such as whole grains, vegetables, fruits, legumes, and nuts) while reducing or limiting the intake of animal-based foods. Eligible exposures included dietary patterns explicitly described as plant-based, pro-vegetarian dietary indices (e.g., plant-based diet index (PDI), healthful PDI (hPDI), unhealthful PDI (uPDI)), the EAT-Lancet planetary health diet, and other composite dietary patterns with a clearly stated plant-forward orientation. These patterns were generally measured using food frequency questionnaires (FFQs), dietary indices, or clustering approaches.

Studies examining isolated dietary components (e.g., intake of olive oil or legumes alone), substitution analyses (e.g., replacing legumes with meat), or focusing on ultra-processed plant-based products (e.g., meat analogs) rather than minimally processed plant foods were excluded. Dietary patterns evaluated only through general healthy eating scores (e.g., DASH, HEI) without a clear plant-based orientation were also excluded, unless the authors explicitly classified them as plant-based.

Although the initial search included the Mediterranean Diet (MD) to ensure comprehensive retrieval of plant-oriented dietary patterns, studies exclusively assessing the MD were excluded during the conceptual refinement phase. The MD does not fully meet our operational definition of PBDs since it incorporates moderate intakes of meat, fish, dairy, and eggs, being strongly shaped by cultural and regional culinary traditions [22,23]. This conceptual distinction has also been recognized in previous methodological papers and reviews, where the MD has been analyzed separately from plant-based indices such as the PDI, hPDI/uPDI, or PVG [16]. Thus, including MD-specific studies in this scoping review would have reduced the internal coherence of our framework.

### 2.3. Study Selection Process

Study selection was carried out in two stages: (1) screening of titles and abstracts, and (2) full-text review. Two authors independently (A.O.-C. and L.C.-A.) performed the screening using the Rayyan platform [24], and any discrepancies were resolved by a third author (I.C). Duplicate records were removed using a reference management tool (Zotero, v.7.0.24). The selection process was documented using the PRISMA-ScR flow diagram [25].

### 2.4. Data Extraction and Synthesis

A structured data extraction form was developed to collect the following information from each study, including the authors and year of publication, the country where the study was conducted, study design, sample size, participants’ characteristics, the dietary pattern evaluated, the type of gastrointestinal cancer examined, and the main findings with corresponding effect estimates.

Data were synthesized using a descriptive and narrative approach, with findings organized by type of dietary patterns and cancer, providing the most important information on study quality parameters. 

### 2.5. Risk of Bias and Limitations

In line with previous recommendations for scoping reviews [20,25,26], we included a table on the risk of bias based on the Newcastle–Ottawa Scale adapted for cohort and case–control studies (Supplementary Materials File S1) and another table in the main material that contained information on the limitations, funding sources, and conflicts of interest reported by the included articles. 

## 3. Results

**Figure 1:**
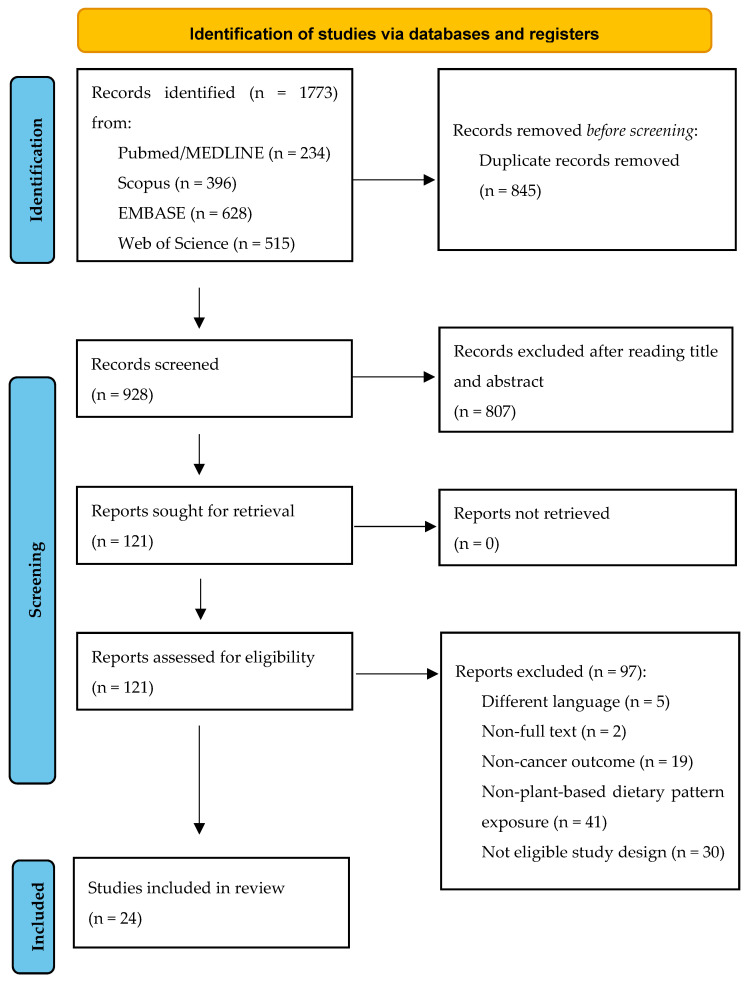
PRISMA flowchart depicting the selection of studies.

The flow diagram following PRISMA guidelines is presented in Figure 1. A total of 1773 studies were initially identified, of which 845 duplicates were removed, leaving 928 references to be examined. Subsequently, titles and abstracts were screened, and after applying exclusion and inclusion criteria, 121 studies were eligible for full-text assessment. After a detailed screening, 24 studies were finally selected for the review, analyzed and summarized in Table 2.

### 3.1. Characteristics of the Included Studies

The main characteristics of the included studies are summarized in Table 2. By geographic region, most studies were performed in Europe (*n* = 10), North America (*n* = 8) and Asia (*n* = 6). By country, the order was as follows: the United States (*n* = 8) [30,33,34,35,39,42,44,47], the United Kingdom (*n* = 6) [27,32,37,41,43,46], Italy (*n* = 3) [15,40,48], Iran (*n* = 3) [29,31,38], China (*n* = 2) [36,45], Malaysia (*n* = 1) [28], and Spain (*n* = 1) [14]. The majority were published in 2023 (*n* = 9) [15,28,29,30,37,39,41,42,47], followed by 2022 (*n* = 5) [14,27,34,35,36], 2024 (*n* = 5) [31,38,40,45,46], 2025 (*n* = 3) [32,43,48], and finally 2021 (*n* = 2) [33,44]. Regarding study design, a total of 16 cohort studies [15,27,30,32,33,34,35,37,39,41,42,43,44,45,46,47] with a follow-up range of 8.8 to 34 years, and eight hospital-based case–control studies [14,28,29,31,36,38,40,48] were included.

### 3.2. PBD Patterns Used in the Included Studies

The FFQ was the main dietary assessment tool used to calculate PBDs in the included studies (*n* = 18). Other dietary assessment methods included 24-hour dietary recalls (*n* = 4) [32,37,43,46], a touchscreen questionnaire (*n* = 1) [27], and a standardized dietary questionnaire (*n* = 1) [45]. The PDI, hPDI, and uPDI were used as reference dietary patterns in 13 studies; however, in one study, only hPDI and uPDI were applied without using the overall PDI [35], while another study only considered the hPDI [46]. Other PBD indices assessed across the studies were the PVGs (gPVG [29], hPVG and uPVG) [14,15], the EAT-Lancet diet with ELD-I [30,32] or PHDI [31], a low-carbohydrate PBD [44], a vegetarian diet [27,41], and a cholesterol-lowering dietary approach [40]. In addition, two studies used ‘a posteriori’ PBDs [28,41], that is, dietary patterns derived from actual consumption data through statistical analysis rather than predefined (Table 2).

### 3.3. Main Results of the Included Studies

As shown in Table 2, there is extensive literature on the reduction in the risk of most digestive tumors in relation to the consumption of PBDs. However, diet quality appears to be particularly relevant, as healthful PBD patterns (hPVG, ELD-I, hPDI) were associated with a lower risk of CRC [27,29,30,31,32,34,35,36,37,38,46,47,48], pancreatic [14,39,40,46,47], HCC [42,43,44,47], esophageal [14,45,46,47,48], stomach [14,46], and oropharyngeal tumors [47,48]. In contrast, unhealthful PBD patterns (uPDI, uPVG) were linked to a higher risk of these same tumors.

Regarding CRC, 13 out of the 15 retrieved studies reported that higher adherence to PBD patterns, particularly healthful ones, was associated with a decreased risk [27,29,30,31,32,34,35,36,37,46,47,48]. Among the two studies that found null associations, one only evaluated the PDI, but not hPDI or uPDI [33], and the other used statistically derived dietary patterns [28]. Notably, of the seven works that examined both the healthful and unhealthful PDIs, all found that higher adherence to the hPDI was a protective factor for CRC, while greater adherence to the uPDI was identified as a risk factor [34,35,36,37,38,47,48] (Table 2).

Pancreatic cancer was investigated in seven studies, of which six reported that higher adherence to PBDs, particularly healthful ones, was associated with a lower risk [14,39,40,46,47,48] (Table 2). The unique study reporting a null association used statistically derived dietary patterns [41]. All the studies that evaluated adherence to the hPDI (4/4) reported a lower pancreatic cancer risk [39,46,47,48], while 2 out of the 3 that evaluated the uPDI reported a higher risk [39,48].

All studies (4/4) investigating HCC reported protective associations [42,43,44,47] (Table 2). One study showed that a low-carbohydrate PBD was protective [44]. The remaining three studies also found protective associations with a higher adherence to the hPDI, although one study found associations only in men [43]. Of the three studies that tested adherence to the uPDI pattern, one reported a higher risk of HCC [43], while the others reported non-significant trends towards higher risk [42,47].

Esophageal cancer was examined in five studies [14,45,46,47,48], of which four reported protective associations with healthful PBD patterns [14,45,46,48]. Out of the four studies that examined the hPDI, three reported a lower risk of esophageal cancer in response to higher adherence [45,46,48].

Stomach cancer was investigated in four studies [14,46,47,48], of which three reported protective associations [14,46,48], two studies using the hPDI [46,48] and one examining the hPVG [14]. Out of the three studies that investigated adherence to unhealthful PBD patterns, all of them reported a higher risk of stomach cancer [14,46,48].

Oropharyngeal cancer was only investigated in two studies, both finding that greater adherence to the hPDI was protective [47,48], while adherence to the uPDI was associated with a higher risk in one study [48] and not associated in the other [47].

Consistent with the associations found for specific localizations, one study reported that the PDI and hPDI were associated with a lower risk of all gastrointestinal cancers [48], while the uPDI was borderline associated with a higher risk [47]. Additionally, another study showed that greater adherence to the gPVG and hPVG patterns was associated with a lower risk of hospitalization due to any type of digestive cancer [15].

### 3.4. Main Limitations of the Included Studies

The limitations, financial support, and conflicts of interest declared by the authors of the included studies are reported in Table 3. Based on the limitations reported by the authors, the most frequently cited limitation was dietary assessment-related measurement error or misclassification due to the use of self-reported tools such as FFQs or 24-hour recalls (*n* = 20) [15,27,28,29,30,31,32,35,36,37,38,39,40,41,43,44,45,46,47,48], followed by the potential for residual or unmeasured confounding inherent to observational designs (*n* = 16) [14,15,27,30,31,32,34,35,37,39,40,41,43,44,46,47]. Small sample size or limited statistical power in subgroup analyses was noted in several studies (*n* = 13) [14,15,27,28,29,30,31,33,34,35,41,42,44]. Limited generalizability of findings, often related to homogeneous study populations (e.g., predominantly White or health professional cohorts), was also commonly reported (*n* = 12) [15,27,30,32,33,35,37,39,40,43,44,47]. Additional limitations included potential selection bias (*n* = 7) [14,28,29,36,38,43,45], recall bias (*n* = 5) [29,36,38,43,48] and baseline-only dietary assessment (*n* = 4) [30,33,39,45]. Most studies reported no conflicts of interest (*n* = 19) [14,15,27,28,29,30,31,32,33,34,36,37,38,39,42,43,45,46,47], while a few studies (*n* = 5) [35,40,41,44,48] reported potential conflicts of interest.

## 4. Discussion

This scoping review mapped the available epidemiological evidence on the association between adherence to PBD patterns and the risk of major digestive cancers. Overall, the evidence consistently supports that greater adherence to PBDs, particularly those emphasizing high-quality and minimally processed plant foods, is associated with a lower risk of most digestive cancers, whereas PBDs rich in refined carbohydrates, sugary foods, and ultra-processed products are associated with a higher risk.

Across prospective cohort and case–control studies conducted in Europe, North America, and Asia, healthful PBD indices, including the hPDI, gPVG, hPVG, the EAT-Lancet diet and plant-based low-carbohydrate or cholesterol-lowering patterns, showed the most consistent protective associations with colorectal, pancreatic, liver, and esophageal cancers, with more limited findings for gastric and oropharyngeal malignancies. In contrast, unhealthful PBD patterns (such as uPDI or uPVG) were positively associated with colorectal, pancreatic, esophageal, and gastric cancers. Although some studies also suggested effect modification by sex, tumor subsite, genetic susceptibility, and lifestyle factors, more studies are needed before firmer conclusions can be drawn.

Our findings are biologically plausible and consistent with previous reviews, where healthful dietary patterns were identified as protective factors, whereas unhealthful patterns were identified as risk factors [49,50,51]. The potential benefits of healthful PBDs may be attributed to a higher intake of fiber, antioxidants, and phytochemicals [6,8], which help reduce systemic inflammation, improve gut microbiota composition [52], and modulate metabolic pathways involved in the carcinogenesis of digestive tumors [53,54]. Conversely, unhealthful PBDs characterized by refined grains, added sugars, and ultra-processed plant foods may increase cancer risk through several mechanisms. These ultra-processed foods are typically rich in fructose, salt, hydrogenated or industrially processed vegetable oils, and food additives such as emulsifiers and artificial sweeteners [55]. These types of products have been associated with an increased risk of several digestive cancers, including colorectal [56], pancreatic [57], liver [58], esophageal [59], and gastric cancer [60]. Specifically, they may promote insulin resistance, oxidative stress, impaired intestinal barrier function, gut dysbiosis, chronic low-grade inflammation, pro-inflammatory signaling, and activation of the PI3K/Akt/mTOR pathway [61,62]. Therefore, the positive associations observed for uPDI indicate that a low intake of animal-derived foods does not necessarily confer protection against digestive cancers. Dietary quality and the degree of food processing appear to be more relevant determinants of cancer risk than the plant or animal origin of food, per se, suggesting that population and primary care recommendations should be made on this premise, supported by previous authors [63].

It is worth noting that, until recently, the most common analytical approach was to evaluate individual food groups, treating them either as risk factors (e.g., red or processed meat) or as protective factors (e.g., legumes) [54]. Indeed, in the latest updates for the primary prevention of CRC, recommendations based on isolated food groups have been progressively replaced by specific dietary patterns, with the hPDI being particularly emphasized [54]. Nevertheless, these analyses and recommendations have focused primarily on CRC, with very limited evidence available for other types of digestive tumors, a gap that this review has helped to address. Actually, our work supports that the protective associations of healthful PBDs do not only apply to CRC (15 studies retrieved), but may also extend to pancreatic (7 studies), esophageal (5 studies), hepatocellular carcinoma (4 studies), stomach (4 studies), and oropharyngeal (2 studies) malignancies. Our findings are supported by previous meta-analyses [10,51,54], including Zhao et al., 2022 [64], which found that plant-based or plant-forward dietary patterns were associated with a lower risk of CRC (25 studies included, adjusted RR = 0.76; 95%CI: 0.69–0.83), while emerging but limited evidence started to mount regarding other digestive cancer localizations including pancreatic cancer (9 cohort studies included, adjusted RR = 0.71; 95%CI: 0.59–0.86), gastric cancer (4 studies included, adjusted RR = 0.81; 95%CI: 0.68–0.97), and HCC (3 studies included, adjusted RR = 0.61; 95%CI: 0.47–0.80).

Beyond cancer prevention, PBDs offer additional benefits related to cardio-metabolic disease prevention [17,18] and environmental sustainability, as they reduce ecological burdens and align with global planetary health goals, such as those proposed by the EAT-Lancet Commission [18]. This positions PBDs as a strategic component of public health policies, integrating chronic disease prevention with environmental impact mitigation. Although sustainability is not a direct risk factor for digestive cancers and health outcomes [65], it is intrinsically linked to dietary quality. Sustainable food systems promote greater availability and consumption of whole plant foods, which are associated with reduced cancer risk, while discouraging reliance on ultra-processed products that may increase risk of all cancers and adverse health outcomes [17,66,67]. Thus, integrating sustainability into dietary recommendations aligns with both cancer prevention and planetary health objectives, consistent with the “One Health” approach.

Despite the clear potential of healthful PBDs, the literature is still scarce due to its novelty. Additionally, substantial heterogeneity was observed across studies in terms of dietary assessment methods and PBD pattern definitions. While some studies performed repeated evaluations, others only assessed diet at a single time point. Although this may not fully capture dietary changes over time, evidence from adult and older populations suggests that overall dietary patterns exhibit moderate to high stability over time, supporting their use as proxies for longer-term exposure [68,69]. Regarding PBD patterns, although 18 of the 24 included studies used FFQs, the length and item structure of these questionnaires varied across studies. However, for plant-based diet indices (PDI, hPDI, uPDI) and pro-vegetarian patterns (gPVG, hPVG, uPVG), the key issue is not the absolute number of items but rather the ability to group foods into the predefined components of each index [6,13]. Thus, FFQs with 70, 90, or 120 items can yield comparable estimates when they systematically cover the food groups (e.g., fruits, vegetables, legumes, whole grains, nuts) that constitute the 12 components of the gPVG/PDI or the 18 components of their complementary healthful and unhealthful scales [8,13]. Overall, this heterogeneity in dietary assessment tools may introduce nondifferential misclassification and attenuate true associations, but it does not invalidate comparability when the food groups required for index computation are adequately represented.

We acknowledge that our scoping review has several limitations. Firstly, evidence selection bias cannot be excluded, as the search was restricted to studies published in English or Spanish, with full-text availability, and published within the last five years, which may have led to the exclusion of evidence published in other languages or time periods. However, this approach allowed us to identify the most relevant evidence and show that healthful PBDs were consistently protective, while unhealthful PBDs were consistently identified as a risk factor for digestive cancer. Secondly, all included studies were observational, making the findings susceptible to residual confounding and measurement error, particularly due to self-reported dietary data. Nevertheless, the fact that consistent associations were observed across studies with different causal and confounding structures in Europe, North America, and Asia argues against this concern.

Our scoping review has several strengths. Firstly, this is one of the first reviews to specifically evaluate how adherence to PBDs, with a focus on dietary quality, influences the risk of digestive neoplasms, providing an up-to-date synthesis of the evidence. By focusing on digestive cancers, this review offers a targeted overview of a heterogeneous and growing field, helping to clarify current research trends and data gaps. Secondly, a systematic, structured, and reproducible search strategy was performed in four databases, and our screening and reporting process followed the methodological recommendations for scoping reviews [19,20,21]. Thirdly, we evaluated the quality of the selected studies, using the Newcastle–Ottawa Scale and reported studies’ limitations, funding, and potential conflicts of interest. Another strength of this review was the identification of important gaps in the literature, such as the limited number of studies conducted in certain geographical areas, the variability in the definition and assessment of PBDs, and the lack of long-term and intervention-based studies. By highlighting these gaps, this review provides valuable guidance for future research.

Future works should include other population groups (children, adolescents, and elders), as well as populations from low- and middle-income countries, to improve the external validity and generalizability of the findings. Future research should also clearly report the dietary components included in the PDI, hPDI, uPDI, and PVG indices, and adopt standardized versions of the EAT-Lancet-based indices (e.g., PHDI or ELD-I), to facilitate comparability across studies and enable the performance of meta-analyses. Finally, given that dietary patterns interact with other risk factors (such as physical inactivity, obesity, alcohol consumption, smoking, and genetic predisposition) to influence digestive cancer risk, comprehensive prevention strategies should integrate dietary guidance with broader lifestyle interventions.

## 5. Conclusions

Overall, the findings of this review highlight that the quality of PBDs is crucial for digestive cancer risk. PBD patterns emphasizing whole and minimally processed plant foods were protective, while those characterized by refined or ultra-processed plant products were deleterious. From a clinical perspective, these findings underscore the importance of prioritizing dietary quality in nutritional counseling, rather than focusing solely on the dichotomy between foods of animal or vegetable origin. Public health implications may differ across population groups: for the general population, encouraging greater consumption of whole plant foods (fruits, vegetables, whole grains, legumes, and nuts) while reducing the intake of red and processed meats appears warranted. For individuals at higher metabolic risk (e.g., those with obesity, type 2 diabetes, or metabolic syndrome), adopting high-quality plant-based patterns (hPDI/hPVG) may offer additional benefits beyond standard dietary recommendations. For those already following vegetarian diets, prioritizing whole over refined plant foods may be more relevant than further restriction of animal products, as current evidence of additional cancer risk reduction beyond overall dietary quality remains limited. Public health policies should consider incorporating plant-forward dietary patterns as part of primary prevention strategies for digestive cancers. Future research should prioritize long-term prospective studies and the development and use of standardized PBD indices to improve comparability, validate the protective effects observed to date, and better inform policy-making.

## Figures and Tables

**Table 1:** Databases, search strategy and number of records retrieved.

Databases	Search Strategy	Results
Pubmed/MEDLINE		
#1	(“diet, plant based” [MeSH Terms] OR “plant-based diet” [All Fields] OR “plant-based index” [All Fields] OR “plant-based indices” [All Fields] OR “PDI” [All Fields] OR “hPDI” [All Fields] OR “uPDI” [All Fields] OR “pro-vegetarian” [All Fields] OR “provegetarian” [All Fields] OR “PVG” [All Fields] OR “hPVG” [All Fields] OR “uPVG” [All Fields] OR “EAT-Lancet diet” [All Fields] OR “diet, mediterranean” [MeSH Terms] OR “Mediterranean diet” [All Fields] OR “diet, vegetarian” [MeSH Terms] OR “vegetarian diet” [All Fields] OR “planetary health diet” [All Fields])	34,132
#2	(“Gastrointestinal Neoplasms” [MeSH Terms] OR “digestive cancer” [All Fields] OR “pancreatic cancer” [All Fields] OR “Pancreatic Neoplasms” [MeSH Terms] OR “oesophagus cancer” [All Fields] OR “Esophageal Neoplasms” [MeSH Terms] OR “stomach cancer” [All Fields] OR “gastric cancer” [All Fields] OR “Stomach Neoplasms” [MeSH Terms] OR “liver cancer” [All Fields] OR “Liver Neoplasms” [MeSH Terms] OR “colorectal cancer” [All Fields] OR “Colorectal Neoplasms” [Mesh])	802,017
#3	#1 AND #2	580
	#1 AND #2 Last 5 years	234
Scopus		
#1	TITLE-ABS-KEY (“plant-based diet” OR “plant-based index” OR “plant-based indices” OR “PDI” OR “hPDI” OR “uPDI” OR “pro-vegetarian” OR “provegetarian” OR “PVG” OR “hPVG” OR “uPVG” OR “EAT-Lancet diet” OR “mediterranean diet” OR “vegetarian diet” OR “planetary health diet”)	50,759
#2	TITLE-ABS-KEY (“gastrointestinal cancer” OR “digestive cancer” OR “pancreatic cancer” OR “esophageal cancer” OR “stomach cancer” OR “gastric cancer” OR “liver cancer” OR “colorectal cancer”)	542,086
#3	#1 AND #2	808
	#1 AND #2 Last 5 years	396
EMBASE		
#1	‘plant based diet’/exp OR ‘plant-based diet’:ti,ab OR ‘plant-based index’:ti,ab OR ‘plant-based indices’:ti,ab OR ‘pdi’:ti,ab OR ‘hpdi’:ti,ab OR ‘updi’:ti,ab OR ‘pro-vegetarian’:ti,ab OR ‘provegetarian’:ti,ab OR ‘pvg’:ti,ab OR ‘hpvg’:ti,ab OR ‘upvg’:ti,ab OR ‘eat-lancet diet’:ti,ab OR ‘mediterranean diet’/exp OR ‘mediterranean diet’:ti,ab OR ‘vegetarian diet’/exp OR ‘vegetarian diet’:ti,ab OR ‘planetary health diet’:ti,ab	42,774
#2	‘gastrointestinal cancer’/exp OR ‘digestive cancer’:ti,ab OR ‘pancreatic cancer’:ti,ab OR ‘pancreatic neoplasm’/exp OR ‘esophageal cancer’:ti,ab OR ‘esophageal neoplasm’ OR ‘stomach cancer’:ti,ab OR ‘gastric cancer’:ti,ab OR ‘stomach neoplasm’/exp OR ‘liver cancer’:ti,ab OR ‘liver neoplasm’/exp OR ‘colorectal cancer’:ti,ab OR ‘colorectal neoplasm’/exp	1,338,833
#3	#1 AND #2	1338
	#1 AND #2 Last 5 years	628
Web of Science		
#1	TS = ((“plant-based diet” OR “plant-based index” OR “plant-based indices” OR “pdi” OR “hpdi” OR “updi” OR “pro-vegetarian” OR “provegetarian” OR “pvg” OR “hpvg” OR “upvg” OR “EAT-Lancet diet” OR “mediterranean diet” OR “vegetarian diet” OR “planetary health diet”))	51,714
#2	TS = ((“gastrointestinal cancer” OR “digestive cancer” OR “pancreatic cancer” OR “esophageal cancer” OR “stomach cancer” OR “gastric cancer” OR “liver cancer” OR “colorectal cancer”))	707,316
#3	#1 AND #2	1067
	#1 AND #2 Last 5 years	515

**Table 2:** Overview of the main characteristics of the studies included in this scoping review.

Location, Author and Year	Design, Study Population and Follow-Up (F-U)	Objective	Dietary Patterns	Main Results	Conclusions
**Colorectal Cancer (CRC)**					
**UK (Watling et al., 2022)** [27]	Cohort (UK Biobank) 5882 CRC cases; age range 40–70 (mean age 56.3 y); 53.9% women. F-U: 11.4 y	To assess the associations of vegetarian and non-vegetarian diets with the risk of all cancers, including CRC.	Touchscreen questionnaire diet groups defined: regular meat-eaters (>5 times/week), low meat-eaters (≤5 times/week), fish-eaters (exclude meat/poultry), vegetarians (exclude all meat and fish, including vegans).	CRC: low meat-eaters HR = 0.91 (95% CI 0.86–0.96) vs. regular meat-eaters. Fish-eaters (HR = 0.84; 0.68–1.03) and vegetarian (HR = 0.78; 0.61; 1.01).	Low meat, fish-based, and vegetarian diets were associated with modestly lower risks of specific cancers.
**Malaysia** **(Abd Rashid et al., 2023)** [28]	Hospital case–control 264 CRC cases: 79 (mean age 61.0 y; 54.4% male): controls: 185 (mean age 50.8 y; 54.1% male). Controls had benign colorectal findings (diverticulum, colitis, etc.). F-U: Not applicable	To determine the main dietary patterns and their association with the risk of CRC in the Malaysian population.	Semi-quantitative FFQ (142 food items). Four dietary patterns were identified using exploratory factor analysis (PBD: fresh and dried fruits, raw and cooked vegetables, spreads; processed diet: confectionery and fast food; energy-dense diet: cereals, alcoholic beverages; allergy diet: condiments, eggs, fish, seafood, dairy products).	The PBD pattern was not significantly associated with CRC risk after adjusting for confounding factors (OR: 1.43 95% CI: 0.69, 2.97 for tertile 2; OR: 0.45 95% CI: 0.19, 1.04 for tertile 3).	No associations were observed between the PBD and the risk of CRC, although those on the third tertile showed a trend towards an inverse association.
**Iran (Nejad et al., 2023)** [29]	Hospital case–control. 213 participants: CRC cases: 71 (mean age 58.2 y; 49.3% male); controls: 142 (mean age 57.7 y; 49.3% male), matched by age and sex. Control diagnoses were non-neoplastic (acute conditions such as fractures). F-U: Not applicable *.	To investigate the association between the gPVG and CRC.	Dietary intake was assessed using a validated semi-quantitative 168-item FFQ. Adherence to the PVG diet was measured using the gPVG.	gPVG: OR (Q3 vs. Q1) = 0.41 (95% CI: 0.18–0.94) and OR (Q4 vs. Q1) = 0.35 (95% CI: 0.14–0.87).	Adherence to a gPVG, characterized by higher intake of vegetables, fruits, cereals, and dairy, and lower meat consumption, was associated with reduced odds of CRC.
**US (Ren et al., 2023)** [30]	Cohort (PLCO) 98,415 participants: CRC cases: 1054; proximal colon cancer cases: 626; distal colon cancer cases: 214; and rectal cancers: 194; non-cases: 97, 361; (mean age 65.5 y); 47.9% male. F-U: 8.8 y	To explore the association between the EAT-Lancet diet and CRC risk.	Dietary information was collected using a validated FFQ with 124 food items. Adherence to the EAT-Lancet diet was quantified using the ELD-I.	ELD-I: HR (Q4 vs. Q1) = 0.81 (95% CI: 0.67–0.98). Although the protective direction was maintained, the associations between ELD-I scores and specific CRC subsites did not reach statistical significance (proximal colon: HR = 0.85; 95% CI: 0.67–1.09; distal colon: HR = 0.73; 95% CI: 0.47–1.12; RC: HR = 0.69; 95% CI: 0.43–1.11).	Higher adherence to the ELD-I score was associated with a lower risk of CRC in a dose–response manner, supporting its potential role in CRC prevention.
**Iran (Mohammadi et al., 2024)** [31]	Hospital case–control. 213 participants: CRC cases: 71 (mean age 58.2 y; 49.3% male); controls: 142 (mean age 57.7 y; 49.3% male), matched by age and sex. Controls were non-neoplastic diagnoses, such as fractures. F-U: Not applicable *.	To assess the association between the PHDI and CRC.	Dietary intake was assessed using a validated semi-quantitative FFQ. Adherence to the EAT-Lancet diet was measured using the PHDI.	PHDI: OR (T3 vs. T1) = 0.41 (95% CI: 0.18–0.91). Total Adequacy Score: OR = 0.26 (95% CI: 0.11–0.62). Total Moderation Score: OR = 0.38 (95% CI: 0.16–0.89). Total Ratio Score: OR = 0.33 (95% CI: 0.13–0.88); no significant trend in third tertile.	Higher PHDI, adequacy, moderation, and ratio scores were associated with lower odds of CRC.
**UK (Hu et al., 2025)** [32]	Cohort (UK Biobank) 177,441 participants: CRC cases: 2016; non-cases: 175,425; aged 37–73 years; 46.3% male. F-U: 13.05 y	To investigate the association between adherence to the EAT-Lancet diet and CRC risk, and to evaluate its interaction with genetic predisposition.	Dietary intake was assessed using the Oxford WebQ 24 h dietary recall questionnaire. Adherence to the EAT-Lancet diet was quantified using the ELD-I. A polygenic risk score (PRS) based on 197 SNPs was used to assess genetic risk.	Higher ELD-I was associated with reduced CRC risk: HR (Q5 vs. Q1) = 0.87 (95% CI: 0.76–0.99). Per SD increase in ELD-I was associated with a 7% reduction in CRC risk (HR: 0.93; 95% CI: 0.89–0.98). High genetic risk (Q5) was associated with a 4.10-fold increased CRC risk (HR: 4.10; 95% CI: 3.48–4.84). Significant additive interaction between ELD-I and PRS: RERI = 0.142 (95% CI: 0.058–0.225); AP = 0.632. Participants with high ELD-I and low PRS had a 75% lower CRC risk compared to those with low ELD-I and high PRS (HR: 0.25; 95% CI: 0.17–0.36).	Greater adherence to the EAT-Lancet diet was associated with reduced CRC risk, particularly among individuals with moderate genetic risk. Diet may offer a feasible and sustainable strategy for CRC prevention, integrating both nutritional and genetic risk factors.
**US (Yue et al., 2021)** [33]	Cohort (NHS II). 94,217 participants: 332 CRC cases; age range 26–45 y (age mean 46.7 y) 100% women. F-U: 24 y	To investigate whether dietary and lifestyle patterns during adulthood are associated with CRC risk overall and by age at diagnosis in younger women from the NHS II.	Repeated FFQs; PDQS, PDI, hPDI, uPDI, and empirical indices of insulinemic potential (EDIH, ELIH).	Hyperinsulinemic patterns increased CRC risk (Q4 vs. Q1: HR: 1.67; 95% CI: 1.15–2.44)), whereas PBD indices showed no association. The overall PDI was non-linearly associated with CRC risk [Q4 vs. Q1: 1.16 (0.81–1.67); Q3 vs. Q1: 1.45 (1.05–2.00)].	Plant-based diet indices were not consistently associated with CRC risk, while higher insulinemic dietary/lifestyle patterns increased risk, especially for early-onset CRC.
**US (Kim et al., 2022)** [34]	Cohort (MEC). 173,427 participants: CRC cases: 4976; non-cases: 168,451; aged 45–75 years; 46% male. Ethnic groups included African American, Japanese American, Latino, Native Hawaiian, and White. F-U: 19.2 y	To evaluate the association between PBD patterns and CRC risk, and to assess whether associations vary by sex, race/ethnicity, and tumor subsite.	Diet was assessed using a validated quantitative FFQ. Three PBD indexes were calculated (range: 18 to 90 points): Overall PDI, hPDI, and uPDI.	Women: No significant associations for any index. No associations across racial/ethnic groups or subsites. Men: PDI: HR = 0.76 (95% CI: 0.67–0.87); hPDI: HR = 0.79 (95% CI: 0.69–0.91); uPDI: HR = 1.08 (95% CI: 0.95–1.22). Subsite-specific: PDI: stronger inverse association for left colon (HR = 0.62) and rectum (HR = 0.69) than right colon (HR = 0.90); hPDI: inverse association across all subsites. uPDI: increased risk for rectal cancer (HR = 1.24)	Greater adherence to PBDs rich in healthy plant foods and low in less healthy plant foods was associated with a reduced CRC risk in men, but not women. The strength of the association varied by race/ethnicity and tumor subsite.
**US (Wang et al., 2022)** [35]	Cohort (NHS and HPFS) 123,733 participants: 3077 CRC cases; age range 30–55 y (NHS) and 40–75 y (HPFS) (mean age 60 y); 61.7% women. F-U: 32 y	To examine the associations of healthy and unhealthy PBDs with the incidence of CRC and its molecular subtypes.	Repeated validated FFQs every 4 years; hPDI and uPDI indices derived (higher hPDI = greater healthy plant food intake; higher uPDI = greater unhealthy plant food intake).	hPDI associated with lower CRC risk (HR = 0.86; 95% CI 0.77–0.96); uPDI associated with higher CRC risk (HR = 1.16; 95% CI 1.04–1.29); hPDI inversely associated with KRAS-wildtype CRC (HR = 0.74; 95% CI 0.57–0.96) but not KRAS-mutant tumors.	Healthy PBDs were associated with a lower CRC risk—especially KRAS-wildtype—while unhealthy PBDs were associated with higher risk.
**China (Wu et al., 2022)** [36]	Hospital case–control. 5598 participants: 2799 CRC cases; 2799 controls; age range: 30–75 y (mean age cases 57.10 y; mean age controls 57.05 y); 42.73% women. F-U: Not applicable *.	To investigate the association between different types of PBD patterns and CRC risk in the Chinese population.	Validated 81-item FFQ; PDI, hPDI and uPDI indices from 16 food groups and analyzed in sex-specific quintiles.	PDI (Q5 vs. Q1) OR = 0.79 (95% CI 0.66–0.95); hPDI OR = 0.45 (95% CI 0.38–0.55); uPDI OR = 1.45 (95% CI 1.18–1.78). In sex-stratified analyses, the inverse PDI association was not observed in women, while the positive uPDI association was not observed in men.	Higher adherence to a healthy PBD was associated with lower CRC risk, whereas higher adherence to an unhealthy PBD was associated with higher risk.
**UK (Liu et al., 2023)** [37]	Cohort (UK Biobank) 186,675 participants: CRC cases: 2163; non-cases: 184,512; aged 37–73 years at baseline; men and women included. Ethnic groups included White, Mixed, Asian Black, Chinese, and Others. F-U: 9.5 y	To investigate the association between PDIs and CRC risk and to explore genetic interaction.	Diet was assessed using the Oxford WebQ, a validated tool that used 24 h recall information and biomarkers. Three PDIs were calculated (ranging from 17 to 85 points–without vegetable oils component): PDI, hPDI y uPDI.	**CRC**: PDI: HR (Q4 vs. Q1) = 0.87 (95% CI: 0.77–0.99); hPDI: HR (Q4 vs. Q1) = 0.85 (95% CI: 0.75–0.97); uPDI: HR (Q2 vs. Q1) = 1.18 (95% CI: 1.04–1.33) and (Q4 vs. Q1) = 1.14 (95% CI: 1.01–1.30). Per 10-point increment in PDI and hPDI: 12% and 9% lower risk of CRC, respectively. **Subsite-specific**: hPDI: HR (Q4 vs. Q1) = 0.77 (95% CI: 0.60–0.98); uPDI: HR (Q4 vs. Q1) = 1.30 (95% CI: 1.02–1.65) for DCC. PDI: HR (Q4 vs. Q1) = 0.76 (0.64–0.91) for RC. In addition, per 10-point increment in PDI and hPDI: 7% and 6% lower risk of RC, respectively. **Genetic risk**: Lowest PRS + highest PDI: HR = 0.41 (95% CI: 0.34–0.50); lowest PRS + highest hPDI: HR = 0.37 (95% CI: 0.30–0.46); highest PRS + highest uPDI: HR = 2.35 (95% CI: 1.92–2.87). No interaction observed between PDIs and PRS.	Greater adherence to higher-quality PDIs was associated with a reduced risk of CRC, particularly for DC and RC. The protective effect was enhanced when combined with lower genetic risk. Diet quality plays a key role in CRC prevention.
**Iran (Yarmand et al., 2024)** [38]	Hospital case–control. 213 participants: 71 CRC cases; 142 controls; age range 40–75 y (mean age cases 58.2 y; mean age controls 57.7 y); 50.7% women. F-U: Not applicable *.	To investigate associations of PBD indices with CRC risk in an Iranian population.	168-item semi-quantitative FFQ; PDI, hPDI and uPDI indices constructed and analyzed in tertiles.	hPDI was inversely associated with CRC risk (T3 vs. T1 OR = 0.21; 95% CI 0.07–0.56) while uPDI was positively associated (T3 vs. T1 OR = 6.76; 95% CI 2.41–18.94); PDI showed no association.	A healthy PBD was associated with a markedly lower risk of CRC, whereas an unhealthy PBD was associated with a substantially higher risk.
**Pancreatic cancer**					
**US** **(Zhong et al., 2023)** [39]	Cohort (PLCO Cancer Screening Trial) 101,748 participants: 421 pancreatic cancer cases; age range 55–74 (mean age 65.5 y); 51.4% female. F-U: 8.86 y	To examine the potential associations of three PBD indices with the risk of pancreatic cancer in a US population.	FFQ with 124 items assessing past year intake; foods grouped into 18 groups then into healthy plant/less-healthy plant/animal foods; PDI/hPDI/uPDI constructed per Satija et al. scoring (quartile scoring).	PDI HR = 0.74 (95% CI 0.57–0.96); hPDI (Q4 vs. Q1) HR = 0.56 (95% CI 0.42–0.75); uPDI HR = 1.38 (95% CI 1.02–1.85). Association of uPDI stronger in BMI < 25 kg/m^2^ subgroup (HR = 3.22; 95% CI 1.56–6.65).	Adherence to a healthful PBD was associated with a lower risk of pancreatic cancer, whereas adherence to an unhealthful PBD was associated with a higher risk.
**Italy (Di Maso et al., 2024)** [40]	Hospital case–control 809 participants: incident pancreatic cancer cases: 258 (median age 63 y; 54.3% male); hospital-based controls: 551 (median age 62 y; 55.2% male), matched by sex, age, and study center. Controls were admitted for acute, non-neoplastic, non-digestive conditions. Participants on lipid-lowering medications were excluded. F-U: Not applicable *.	To evaluate the association between adherence to a plant-based cholesterol-lowering diet (CLD) and the risk of pancreatic cancer.	A 7-point CLD score derived from a validated 78-item FFQ.	High adherence to the CLD (score 5–7) was associated with a 70% reduced risk of pancreatic cancer compared to low adherence (score 0–2): OR = 0.30 (95% CI: 0.18–0.52). The inverse association was strongest among individuals with low or medium physical activity.	Greater adherence to a plant-based CLD was inversely associated with pancreatic cancer risk. The effect was modified by physical activity levels, with stronger associations observed in sedentary individuals.
**UK (Shyam et al., 2023)** [41]	Cohort (UK Women’s Cohort Study) 35,365 participants: pancreatic cancer cases: 136; non-cases: 35,229; mean age at enrollment 52 y, 9 SD; only women included. F-U: 18.5 y	To evaluate the associations between dietary patterns and pancreatic cancer risk.	Diet was assessed using a 217-item FFQ based on the EPIC questionnaire used for vegetarians. Dietary patterns include: 1. Self-reported vegan/vegetarian diets; 2. Diet quality indices (WHO-HDI); 3. Mediterranean Diet score and; 4. Five patterns selected using principal component analysis: prudent; meat-based; fast food–SSB–carbohydrate-rich snacks; ready-to-eat cereal and dairy-rich; and low-diversity–low-fat.	Self-reported vegan or vegetarian diet: not significantly associated (HR = 1.16; 95% CI: 0.76–1.79). WHO-HDI score: not significantly associated (per unit increase, HR = 0.98; 95% CI: 0.90–1.07). Posteriori (data-derived) dietary patterns: “Prudent” pattern: HR = 0.99 (95% CI: 0.92–1.07). “Meat-based” pattern: HR = 1.02 (95% CI: 0.94–1.11). “Fast food, sugary drinks, snacks” pattern: HR = 0.98 (95% CI: 0.88–1.09). “Cereal and dairy-rich” pattern: HR = 1.04 (95% CI: 0.95–1.15). “Low-diversity, low-fat” pattern: HR = 1.02 (95% CI: 0.91–1.15).	Commonly consumed dietary patterns, including those of low dietary quality, were not associated with pancreatic cancer risk over long-term follow-up.
**Hepatocellular cancer (HCC)**					
**US (Kim et al., 2023b)** [42]	Cohort (MEC) 170,321 participants: HCC cases: 722; non-cases: 169,599; aged 45–75 years (mean age 59.5 y, SD 8.8); 46.3% male. Ethnic groups included African American, Japanese American, Latino, Native Hawaiian, and White. F-U: 19.6 y	To investigate the association between PBD patterns and HCC risk, and to assess whether associations vary by sex or race/ethnicity.	Diet was assessed using a validated quantitative FFQ. Three PBD indexes were calculated (range: 18 to 90 points): Overall PDI, hPDI, and uPDI.	Per 10-point increase: PDI: HR = 0.82 (95% CI: 0.71–0.94; hPDI: HR = 0.84 (95% CI: 0.74–0.96); uPDI: HR = 1.08 (95% CI: 0.95–1.23). Participants in the highest quintile (Q5) of PDI had a 23% lower risk of HCC compared to Q1 (HR = 0.77; 95% CI: 0.61–0.98). For hPDI, Q5 vs. Q1 showed a 19% lower risk (HR = 0.81; 95% CI: 0.63–1.03). No significant heterogeneity across racial/ethnic groups.	Greater adherence to a PBD rich in healthy plant foods and low in less healthy plant foods was associated with a reduced risk of HCC. The quality of plant-based foods is crucial, and the benefits were more evident among never smokers.
**UK (Dong et al., 2025)** [43]	Cohort (UK Biobank) 187,781 participants: HCC cases: 177; non-cases: 187,604; aged 37–73 years (mean age: males 56.4 y, SD 8.0; females 55.4 y, SD 7.8); 48.1% male. F-U: 16 y	To investigate the association between PBD patterns and the risk of HCC incidence and liver disease mortality.	Dietary intake was assessed using the Oxford WebQ 24 h dietary recall questionnaire. Three PBD indexes were constructed, based on 17 food groups and scored using quintile-based positive and reverse scoring (range: 17–85 points): PDI, hPDI, and uPDI.	In males, higher hPDI scores were associated with lower HCC incidence (HR (Q5 vs. Q1): 0.47; 95% CI: 0.26–0.85) and lower liver disease mortality (HR: 0.46; 95% CI: 0.27–0.77). Higher uPDI scores were associated with increased HCC incidence (HR: 1.90; 95% CI: 1.00–3.63) and liver disease mortality (HR: 2.21; 95% CI: 1.37–3.57). No significant associations were found in females.	Greater adherence to a hPDI may reduce the risk of HCC and liver disease mortality in males, but not in females. The quality of plant-based foods is crucial in cancer disease prevention.
**US (Liu et al., 2021)** [44]	Cohort (NHS and HPFS) 136,967 participants: HCC cases: 156; non-cases: 136,811; aged 30–55 years at baseline in NHS (females) and 40–75 years at baseline in HPFS (males); 35.2% male. F-U: 26.7 y	To examine the association between three Low-Carb Diet (LCDs) scores and HCC risk and assess the relationship of total, animal and plant-origin macronutrient intake with HCC risk.	Dietary data were collected using a validated semi-quantitative FFQ every 4 years. Three LCDs were calculated: an overall LCD, an animal-based LCD and a plant-based LCD (scoring positive the consumption of fats and proteins, and conversely, the carbohydrate consumption).	Plant-based LCD: HR per 1 SD increment = 0.83 (95% CI: 0.70–0.98); overall LCD and animal-based LCD: no significant associations. Higher intake of plant fat was associated with lower HCC risk (HR per 1 SD increment = 0.78; 95% CI: 0.65–0.95), while refined grain carbohydrates were associated with higher risk (HR = 1.18; 95% CI: 1.00–1.39). Isocaloric substitution of 5% energy from carbohydrates or refined grains with plant fat and protein was associated with 26% (HR = 0.74; 95% CI: 0.58–0.93) and 30% (HR = 0.70; 95% CI: 0.55–0.90) lower risk of HCC, respectively.	Greater adherence to a plant-based LCD and lower intake of refined grain carbohydrates was associated with lower risk of HCC. Replacing carbohydrates, particularly from refined grains, with plant fat and protein may further enhance the protective association. Macronutrient quality and source are key factors in HCC prevention.
**Esophageal cancer**					
**China (Zhang et al., 2024)** [45]	Cohort (NCEDTP) 15,184 participants: 176 esophageal cancer cases; age range 40–69 (mean age 51.8 y); 54% female. F-U: 17 y	To examine the associations between three predefined indices of PBD patterns and the risk of esophageal cancer.	Structured FFQ (range 15–75 points; 15 food groups adapted to local diet). Indices: PDI, hPDI, and uPDI.	hPDI (Q4 vs. Q1) HR = 0.50 (95% CI 0.32–0.77); per 10-point increase HR = 0.42 (95% CI 0.27–0.66). uPDI Q4 vs. Q1 HR = 1.80 (95% CI 1.16–2.82); per 10-point increase HR = 1.90 (95% CI 1.26–2.88). Overall, PDI showed a non-significant inverse trend.	A healthful PBD was associated with a substantially lower risk of esophageal cancer, whereas an unhealthful PBD was associated with a higher risk.
**Several gastrointestinal cancer localizations**					
**UK** **(Cai et al., 2024)** [46]	Cohort 105,463 participants: GIC cases: 1661; non-cases: 103,802; aged 40–72 years (mean 56.6 y, SD 7.8); 48.1% male. F-U: 11.7 y	To evaluate how healthy dietary patterns (including hPDI) and genetic predisposition contribute independently and jointly to the risk of GIC.	24 h dietary recall questionnaire (Oxford WebQ). hPDI (18–90 points, 18 components).	Association with GIC risk after full adjustment. **Colorectal**: hPDI: HR = 0.73 (95% CI: 0.61–0.87). **Pancreatic**: hPDI: HR = 0.36 (95% CI: 0.24–0.54). **Esophageal**: hPDI: HR = 0.62 (95% CI: 0.39–0.97). **Gastric**: hPDI: HR = 0.51 (95% CI: 0.30–0.88).	Greater adherence to hPDI was significantly associated with lower risk of all four GICs.
**US (Kim et al., 2023a)** [47]	Cohort (NHS, NHS II, HPFS) 211,673 participants: digestive system cancer cases: 6518; non-cases: 205,155; aged 25–75 years (mean age 58 y); men and women included. F-U: NHS (34 y), NHS II (26 y), HPFS (30 y).	To examine the associations between three predefined PBD indices and the risk of total and site-specific digestive system cancers.	Dietary intake was assessed using validated semi-quantitative FFQs every 4 years. Three PBD indexes were calculated (ranging from 18 to 90 points): Overall PDI, hPDI, and uPDI.	**Total digestive system cancer**: PDI: HR = 0.94 (95% CI: 0.89–0.99); hPDI: HR = 0.93 (95% CI: 0.89–0.97); uPDI: HR = 1.04 (95% CI: 0.99–1.08). **Oropharyngeal cancer**: PDI: HR = 0.78 (95% CI: 0.65–0.95); hPDI: HR = 0.86 (95% CI: 0.73–1.02); uPDI: HR = 0.97 (95% CI: 0.83–1.13). **Esophageal cancer**: PDI: HR = 1.09 (95% CI: 0.86–1.40); hPDI: HR = 0.91 (95% CI: 0.74–1.12); uPDI: HR = 1.12 (95% CI: 0.91–1.37). **Stomach cancer**: PDI: HR = 1.22 (95% CI: 0.97–1.54); hPDI: HR = 1.10 (95% CI: 0.90–1.35); uPDI: HR = 0.98 (95% CI: 0.81–1.19). **Small intestine cancer**: PDI: HR = 0.86 (95% CI: 0.60–1.24); hPDI: HR = 0.86 (95% CI: 0.63–1.18); uPDI: HR = 0.87 (95% CI: 0.65–1.17). **CRC**: PDI: HR = 0.98 (95% CI: 0.92–1.05); hPDI: HR = 0.94 (95% CI: 0.89–1.00); uPDI: HR = 1.07 (95% CI: 1.01–1.13). CC: PDI: HR = 0.98 (95% CI: 0.90–1.07); hPDI: HR = 0.94 (95% CI: 0.87–1.01); uPDI: HR = 1.04 (95% CI: 0.97–1.11). **RC**: PDI: HR = 1.02 (95% CI: 0.88–1.19); hPDI: HR = 0.99 (95% CI: 0.87–1.13); uPDI: HR = 1.08 (95% CI: 0.96–1.22). **Pancreatic cancer**: PDI: HR = 0.83 (95% CI: 0.73–0.94); hPDI: HR = 0.91 (95% CI: 0.81–1.01); uPDI: HR = 0.93 (95% CI: 0.84–1.04). **Biliary tract cancer**: PDI: HR = 1.01 (95% CI: 0.77–1.32); hPDI: HR = 0.98 (95% CI: 0.78–1.23); uPDI: HR = 1.04 (95% CI: 0.84–1.29). **HCC**: PDI: HR = 0.69 (95% CI: 0.50–0.95); hPDI: HR = 0.68 (95% CI: 0.52–0.91); uPDI: HR = 1.08 (95% CI: 0.83–1.40).	A hPDI was associated with a lower risk of total digestive system cancers, particularly colorectal, liver, and pancreatic cancers. In contrast, the uPDI was associated with an increased risk of colorectal and gastrointestinal tract cancers. The quality of plant-based foods is crucial in cancer prevention.
**Italy (Turati et al., 2025)** [48]	Hospital case–control. Oral/pharyngeal cancer: 942 cases (752 men; 190 women; median age 58 y) and 2492 controls (1497 men; 995 women; median age 58 y); esophageal cancer: 304 cases (275 men; 29 women; median age 60 y) and 743 controls (593 men; 150 women; median age 60 y); stomach cancer: 230 cases (143 men; 87 women; median age 63 y) and 547 controls (286 men; 261 women; median age 63 y); CRC: 1953 cases (1125 men; 828 women; median age 62 y) and 4154 controls (2073 men; 2081 women; median age 58 y); pancreatic cancer: 326 cases (174 men; 152 women; median age 63 y) and 652 controls (348 men; 304 women; median age 63 y). F-U: Not applicable *.	To examine the association of the overall PDI, hPDI and uPDI with the risk of selected digestive cancers.	Validated interviewer-administered FFQ on usual diet 2 years before diagnosis/admission; PBD indices (PDI, hPDI, uPDI) from 16 food groups (healthy plant, unhealthy plant, animal foods).	**Oropharyngeal**: PDI was inversely associated (Q5 vs. Q1) OR = 0.63; 95% CI 0.47–0.84) **Esophageal**: PDI was inversely associated (T3 vs. T1) OR = 0.47; 95% CI 0.31–0.7). hPDI inversely associated with all sites (e.g., CRC OR = 0.69; 95% CI 0.57–0.84). uPDI positively associated with oral/pharyngeal (OR (Q5 vs. Q1) =1.43, 95% CI 1.06–1.94), colorectal (OR = 2.28, 95% CI 1.86–2.81), and pancreatic cancer (OR = 1.74, 95% CI 1.14–2.65)	A healthy PBD was associated with a lower risk of several digestive cancers, whereas an unhealthy PBD increased the risk.
**Spain (Oncina-Cánovas et al., 2022)** [14]	Hospital case–control (PANESOES). 1233 participants: esophageal cancer cases: 199 (mean age 60.5 y; 92.5% male); stomach cancer cases: 414 (mean age 64.8 y; 65.5% male); pancreatic cancer cases: 165 (mean age 65.2 y; 60.6% male); hospital-based controls: 455 (mean age 63 y; 62.6% male), matched by age, sex and province. Control diagnoses were a priori unrelated to the exposure of interest (e.g., hernias). F-U: Not applicable *.	To investigate the association between three PVG food patterns and cancers of esophagus, stomach and pancreas.	Dietary data were collected using a validated semi-quantitative 93-item FFQ based on the Harvard questionnaire. Three PVG food patterns were calculated: the gPVG, the hPVG and the uPVG.	**Pancreatic**: gPVG: RRR (Q5 vs. Q1) = 0.43 (95% CI: 0.35–0.52); hPVG: RRR = 0.74 (95% CI: 0.59–0.92). **Esophageal**: gPVG: RRR (Q5 vs. Q1) = 0.37 (95% CI: 0.32–0.42); hPVG: RRR = 0.72 (95% CI: 0.58–0.90). **Stomach**: gPVG: RRR (Q5 vs. Q1) = 0.34 (95% CI: 0.27–0.43); hPVG: RRR = 0.42 (95% CI: 0.34–0.52); uPVG: RRR = 1.76 (95% CI: 1.42–2.18). uPVG: No significant associations were observed for esophagus cancer and pancreatic cancer.	Greater adherence to gPVG and hPVG was associated with a lower risk of esophagus, stomach and pancreatic cancer, while greater adherence to uPVG was associated with a higher risk of stomach cancer. The quality of plant-based foods is essential for effective dietary guidance in the prevention of digestive tumors.
**Italy (Martinez et al., 2023)** [15]	Cohort (Moli-Sani Study) 24,325 participants: digestive cancer cases: 598; non-cases: 23,727; aged ≥35 years (mean age 55.2 y, 11.7 SD); 48% male. F-U: 12.9 y	To examine the association between three PVG food patterns and cancer risk (including digestive cancers).	Dietary data were collected using a semi-quantitative FFQ (EPIC model). Three PVG food patterns were calculated: the general PVG pattern (gPVG), the healthful PVG pattern (hPVG) and the unhealthful PVG pattern (uPVG).	**Hospitalization due to any cancer:** gPVG: HR (Q5 vs. Q1) = 0.85 (95% CI: 0.75–0.97); per 1 SD increment: HR = 0.95 (95% CI: 0.91–0.99). No associations were observed for hPVG or uPVG patterns with overall cancer risk. **Hospitalization risk due to any digestive cancer:** gPVG pattern: associated with lower hospitalization rates for digestive cancers (HR Q5 vs. Q1 = 0.74; 95% CI: 0.57–0.95); hPVG: inversely associated with digestive cancer hospitalizations (HR Q5 vs. Q1 = 0.76; 95% CI: 0.58–0.99); uPVG: positively associated with respiratory cancer hospitalizations (HR Q5 vs. Q1 = 1.68; 95% CI: 1.06–2.68).	Greater adherence to a gPVG pattern, rich in plant-based foods and low in animal products, was associated with lower cancer risk assessed through cancer hospitalizations. These findings highlight the importance of both plant food quality and minimal animal product consumption in cancer prevention.

*In alphabetical order:* AHEI-2010: Alternative Health Eating Index-2010; CC: colon cancer; CLD: cholesterol-lowering diet; CRC: colorectal cancer; DCC: distal colon cancer; ELD-I: EAT-Lancet Diet Index; FFQ: food frequency questionnaire; GIC: gastrointestinal cancer; HDI: Healthy Diet Indicator; hPDI: healthful plant-diet index; HPFS: Health Professionals Follow-up Study; HR: Hazard Ratio; MedDiet: Mediterranean Diet; NHS: Nurses’ Health Study; NHS II: Nurses’ Health Study II (NHS II); PBD: plant-based diet/dietary; PCC: proximal colon cancer; PDI: overall plant-diet index; PHDI: planetary health diet index; PRS: polygenic risk score; RC: rectal cancer; RERI: Relative Excess Risk Due to Interaction; SD: Standard Deviation; SNPs: Single-Nucleotide Polymorphisms; uPDI: unhealthful plant-diet index; UK: United Kingdom; US: United States. * Not applicable: due to the retrospective design of the study, follow-up information is not applicable as it is in prospective studies.

**Table 3:** Limitations and information on funding and conflicts of interest declared in the studies.

Author and Year	Main Limitations	Funding/Support	Conflicts of Interest
**Liu et al. (2021)** [44]	Limited number of HCC cases affects risk estimate accuracy; confounding bias because it was not possible to adjust for all comorbidities and liver diseases; HBV/HCV data available only for a small group; the participants were predominantly White, educated health professionals, which may limit generalizability to other populations; observational design allows possible residual confounding; FFQs are tools that are prone to measurement error.	Supported by UM1 CA186107 (Nurses’ Health Study infrastructure grant), P01 CA87969 (Nurses’ Health Study program grant for cancer research), U01 CA167552 (Health Professionals Follow-up Study infrastructure grant), NIH K24 DK098311 (ATC), and NIH K07 CA188126 (XZ), R21CA238651 (XZ), American Cancer Society Research Scholar Grant (RSG NEC-130476, XZ). XZ is also supported by the Dana-Farber Harvard Cancer Center (DF/HCC) GI SPORE Developmental Research Project Award (P50CA127003) and DF/HCC Nodal Award (Cancer Center Support Grant, P30CA006516-55). ATG is a Stuart and Suzanne Steele MGH Research Scholar.	One author (JAM) declared to have received research funding from Boston Biomedical and acted as a consultant for several companies. Other authors declared no conflicts of interest.
**Yue et al. (2021)** [33]	Lack of genetic data; small number of CRC cases (especially early-onset); limited generalizability (mainly White women).	NIH (U01CA176726, R37CA246175, R00CA215314), American Cancer Society (MRSG-17-220-01-NEC).	The authors declared no conflicts of interest.
**Kim et al. (2022)** [34]	Diet assessed only at baseline (updated for 46% of participants); potential residual confounding; negative scoring of all animal foods may overlook potential benefits of fish and dairy; limited power in subgroup analyses (e.g., Native Hawaiians).	Supported by the National Research Foundation of Korea (2021R1A2C1003211) and the U.S. National Cancer Institute (R03 CA223890, U01 CA164973).	The authors declared no conflicts of interest.
**Oncina-Cánovas et al. (2022)** [14]	Small sample size, particularly for esophageal and pancreatic cancer, reducing statistical power; case–control design is more susceptible to biases, including selection bias; diet was assessed five years prior to the interview, possibly leading to misclassification; potential confounders may not have been accounted for in the analysis.	Funded by the Spanish Ministry of Health (FIS 91/0435, RCESP C 03/09), Generalitat Valenciana (EVES 030/2005, CTGCA/2002/06, G03/136), and CIBERESP.	The authors declared no conflicts of interest.
**Wang et al. (2022)** [35]	Potential unmeasured or residual confounding; dietary data from questionnaires (measurement error); limited tissue availability for molecular analyses; small subgroups reduced power; limited generalizability (mostly White health professionals).	NIH (multiple grants), Cancer Research UK, Japan Society for the Promotion of Science, Prevent Cancer Foundation.	ATC investigator for Zoe Global Ltd.; MG received funding from pharma; JAM consultancy for COTA Healthcare; others declared no conflicts of interest.
**Watling et al. (2022)** [27]	Limited cancer cases among vegetarians/fish-eaters; incomplete cancer registry data after 2015/2019; inability to adjust for total energy intake; possible residual confounding, misclassification, and chance findings; limited generalizability (mostly White British).	Cancer Research UK (C8221/A29017), World Cancer Research Fund (2019/1953), Wellcome Trust, MRC, University of Oxford, other fellowships.	The authors declared no conflicts of interest.
**Wu et al. (2022)** [36]	Recruitment from a single hospital (potential selection bias); recall bias; questionnaire lacked key food items (e.g., oils, coffee, tea, animal fats).	National Natural Science Foundation of China, Guangdong Basic and Applied Research Foundation.	The authors declared no conflicts of interest.
**Abd Rashid et al. (2023)** [28]	The FFQ, although validated, relies on self-reporting; the sample is hospital-based and may not be representative of the entire Malaysian population; limited sample size, especially in some subgroups (e.g., Indian ethnicity not present in cases).	The study is funded by the Long-Term Research Grant Scheme (LRGS)-Malaysia Research University Network (MRUN) (203/PPSK/6720021, LRGS/MRUN/FI/02/2018/01, and LR001-2019).	The authors declared no conflicts of interest.
**Kim et al. (2023a)** [47]	Residual confounding cannot be ruled out; limited generalizability due to predominantly White health professional population; inability to assess strict vegetarian or vegan diets; potential misclassification of dietary intake.	Supported by the National Research Foundation of Korea (Grant No. 2021R1A2C1003211) and the U.S. National Institutes of Health (NIH) (Grant Nos. UM1 CA186107, P01 CA87969, U01 CA176726, U01 CA167552).	The authors declared no conflicts of interest.
**Kim et al. (2023b)** [42]	Lack of data on HBV/HCV infection and cirrhosis; dietary updates only available for 46% of participants; limited power in race-specific analyses due to small case numbers.	Supported by the National Research Foundation of Korea (2021R1A2C1003211) and the U.S. National Cancer Institute (R01 CA228589, U01 CA164973, R03 CA223890).	The authors declared no conflicts of interest.
**Liu et al. (2023)** [37]	Low participation rate (5.5%) in UK Biobank may introduce selection bias and therefore, lack of representativeness may distort genetic associations; the use of 24-hour recall for dietary assessment is prone to measurement errors leading to misclassification; only 17 food groups included in PDIs due to lack of data on vegetable oils; PDIs treat all animal foods equally, ignoring potential benefits of some groups, such as dairy and seafood; no distinction between red and white meat (different association with CRC); possible residual confounding; analyses limited to European populations.	Funded by the National Key R&D Program of China (2021YFC2500400, 2021YFC2500401), National Natural Science Foundation of China (81974488), Tianjin Key Medical Discipline Construction Project (TJYXZDXK-009 A), the Young Elite Scientists Sponsorship Program by China Association for Science and Technology (YESS20210143) and Guangdong Basic and Applied Basic Research Foundation (2022A1515010436)	The authors declared no conflicts of interest.
**Martinez et al. (2023)** [15]	Possible residual confounding due to the observational design; only cancer hospitalizations considered, possibly missing outpatient cases; risk estimates may be limited by small subgroup events; no repeated dietary assessments; self-reported diet data prone to error; classification of healthy plant foods may be subjective; potatoes scored unhealthy regardless of preparation; generalizability limited to the Italian population.	Partially funded by the Italian Ministry of Health (Ricerca Corrente 2022–2024).	The authors declared no conflicts of interest.
**Nejad et al. (2023)** [29]	Presence of selection bias; possible recall bias due to FFQs; long-term effects of risk factors on CRC could not be evaluated; hospital-based design was another limitation; relatively small sample size.	Not reported.	The authors declared no conflicts of interest.
**Ren et al. (2023)** [30]	Diet was assessed only once at baseline, without accounting for dietary changes over time; potential residual confounding cannot be excluded; limited CRC subsite cases reduced statistical power; results may not be generalizable beyond older American adults; no significant subgroup interactions observed.	Supported by the General Project of Chongqing Natural Science Foundation, Chongqing Science and Technology Commission, China [cstc2021jcyjmsxmX0153 (LP)], [cstc2021jcyj-msxmX0112 (YW)], [CSTB2022NSCQ-MSX1005 (HG)], and the Kuanren Talents Project of the Second Affiliated Hospital of Chongqing Medical University in China [kryc-yq-2110 (HG)].	The authors declared no conflicts of interest.
**Shyam et al. (2023)** [41]	Low number of pancreatic cancer cases may have limited statistical power; dietary intake and physical activity were self-reported, introducing potential measurement error; FFQs did not capture key dietary details (e.g., cooking methods, additives); lack of data on specific fatty acids affected score accuracy; the relatively healthy cohort may have diluted associations; residual confounding cannot be ruled out.	Funded by the Institute for Research, Development and Innovation at the International Medical University [IMU 435/2019].	One author (JD) declared to be the Director of Dietary Assessment Ltd. Other authors declared no conflicts of interest.
**Zhong et al. (2023)** [39]	PDI not specific to pancreatic cancer; baseline-only diet assessment; possible nondifferential bias from changes in lifestyle; limited generalizability (mostly non-Hispanic White, lower education, aspirin users, smokers); residual confounding.	National Natural Science Foundation of China, China Postdoctoral Science Foundation, Chongqing Postdoctoral Research Funding.	The authors declared no conflicts of interest.
**Cai et al. (2024)** [46]	Use of a single 24 h dietary recall may introduce measurement error; potential residual confounding; CRC not stratified by molecular subtype or staging.	NSFC-82130098 (China), Fundamental Research Funds for the Central Universities (WHU:2042022kf1205), Wuhan Knowledge Innovation Program (whkxjsj011).	The authors declared no conflicts of interest.
**Di Maso et al. (2024)** [40]	Potential recall bias from self-reported dietary data; lack of adjustment for obesity due to possible misclassification; limited generalizability outside Mediterranean populations; dietary cut-offs derived from the study population; potential residual confounding.	Partially funded by the Italian Ministry of Health (Ricerca Corrente).	One author (DJAJ) reported numerous research grants and honoraria from the food industry and health-related organizations. Other authors declared no conflicts of interest.
**Mohammadi et al. (2024)** [31]	Small sample size; possible unmeasured confounders; use of FFQs relies on memory and may lead to measurement errors; hospital-based participant selection may introduce selection bias.	Not reported.	The authors declared no conflicts of interest.
**Yarmand et al. (2024)** [38]	Selection bias and recall bias inherent to the case–control design.	Not reported.	The authors declared no conflicts of interest.
**Zhang et al. (2024)** [45]	PDI classification based on local dietary habits; non-quantitative FFQ (frequency, not servings); no energy intake adjustment; diet measured only at baseline (possible misclassification); missing lifestyle data; 49% compliance with screening (selection bias).	Beijing Nova Program, CAMS Innovation Fund, Cancer Hospital Talent Incentive Program.	The authors declared no conflicts of interest.
**Dong et al. (2025)** [43]	Potential recall bias from dietary assessments; lack of data on food preparation methods; residual confounding; observational design limits causal inference; limited generalizability beyond White European populations.	Supported by the Natural Science Foundation of Guangdong Province, China (grant number 2022A1515011744) and the Science and Technology Program of Guangzhou, China (grant number 202201011485).	The authors declared no conflicts of interest.
**Hu et al. (2025)** [32]	Potential residual confounding due to observational design; reliance on self-reported dietary data; limited compatibility of ELD-I with 24 h recall format; lack of data on Lynch syndrome; generalizability limited to European ancestry.	Supported by the Outstanding Scientific Fund of Shengjing Hospital (Qi-Jun Wu).	The authors declared no conflicts of interest.
**Turati et al. (2025)** [48]	Possible selection bias; recall bias and dietary measurement error; retrospective design allows reverse causation; incomplete FFQ coverage (e.g., juices, animal-based foods, nuts); results may not reflect current dietary patterns.	AIRC Foundation (IG 21378); PRIN 2022 (2022A4WZFC); Italian Ministry of Health (Ricerca Corrente).	LSAA member and CLV member of ICQC; LSAA received honoraria from the Nutrition Foundation of Italy; other authors declared no conflicts of interest.

## Data Availability

The original contributions presented in this study are included in the article and Supplementary Materials. No new data were created in this study.

## Supplementary Materials

The following supporting information can be downloaded at: https://www.mdpi.com/article/10.3390/nu18050756/s1. Table S1: Risk of bias assessment using Newcastle-Ottawa Scale adapted for case-control studies; Table S2: Risk of bias assessment using Newcastle-Ottawa Scale adapted for cohort studies.

## Abbreviations

The following abbreviations are used in this manuscript:

EDIH	Empirical Dietary Index for Hyperinsulinemia
ELIH	Empirical Lifestyle Index for Hyperinsulinemia
FFQ	Food frequency questionnaire
HCC	Hepatocellular carcinoma
hPDI	Healthful plant-based diet index
hPVG	Healthful pro-vegetarian pattern
LCD	Low-Carb Diet
MD	Mediterranean Diet
PBD	Plant-based dietary
PDI	Plant-based diet index
PDQS	Plant-based diet quality score
PVG	Pro-vegetarian
uPDI	Unhealthful plant-based diet index
uPVG	Unhealthful pro-vegetarian pattern

## References

[1] Bray F., Laversanne M., Sung H., Ferlay J., Siegel R.L., Soerjomataram I., Jemal A. (2024). Global Cancer Statistics 2022: GLOBOCAN Estimates of Incidence and Mortality Worldwide for 36 Cancers in 185 Countries. CA A Cancer J. Clin..

[2] Li S., He Y., Liu J., Chen K., Yang Y., Tao K., Yang J., Luo K., Ma X. (2024). An Umbrella Review of Socioeconomic Status and Cancer. Nat. Commun..

[3] Key T.J., Bradbury K.E., Perez-Cornago A., Sinha R., Tsilidis K.K., Tsugane S. (2020). Diet, Nutrition, and Cancer Risk: What Do We Know and What Is the Way Forward?. BMJ.

[4] Ruze R., Song J., Yin X., Chen Y., Xu R., Wang C., Zhao Y. (2023). Mechanisms of Obesity- and Diabetes Mellitus-Related Pancreatic Carcinogenesis: A Comprehensive and Systematic Review. Signal Transduct. Target. Ther..

[5] Brauer M., Roth G.A., Aravkin A.Y., Zheng P., Abate K.H., Abate Y.H., Abbafati C., Abbasgholizadeh R., Abbasi M.A., Abbasian M. (2024). Global Burden and Strength of Evidence for 88 Risk Factors in 204 Countries and 811 Subnational Locations, 1990–2021: A Systematic Analysis for the Global Burden of Disease Study 2021. Lancet.

[6] Tapsell L.C., Neale E.P., Satija A., Hu F.B. (2016). Foods, Nutrients, and Dietary Patterns: Interconnections and Implications for Dietary Guidelines. Adv. Nutr..

[7] Tessier A.-J., Wang F., Korat A.A., Eliassen A.H., Chavarro J., Grodstein F., Li J., Liang L., Willett W.C., Sun Q. (2025). Optimal Dietary Patterns for Healthy Aging. Nat. Med..

[8] Storz M.A. (2022). What Makes a Plant-Based Diet? A Review of Current Concepts and Proposal for a Standardized Plant-Based Dietary Intervention Checklist. Eur. J. Clin. Nutr..

[9] Melina V., Craig W., Levin S. (2016). Position of the Academy of Nutrition and Dietetics: Vegetarian Diets. J. Acad. Nutr. Diet..

[10] Bai T., Peng J., Zhu X., Wu C. (2023). Vegetarian Diets and the Risk of Gastrointestinal Cancers: A Meta-Analysis of Observational Studies. Eur. J. Gastroenterol. Hepatol..

[11] Greenwell J., Grant M., Young L., Mackay S., Bradbury K.E. (2024). The Prevalence of Vegetarians, Vegans and Other Dietary Patterns That Exclude Some Animal-Source Foods in a Representative Sample of New Zealand Adults. Public. Health Nutr..

[12] Martínez-González M.A., Sánchez-Tainta A., Corella D., Salas-Salvadó J., Ros E., Arós F., Gómez-Gracia E., Fiol M., Lamuela-Raventós R.M., Schröder H. (2014). A Provegetarian Food Pattern and Reduction in Total Mortality in the Prevención Con Dieta Mediterránea (PREDIMED) Study. Am. J. Clin. Nutr..

[13] Satija A., Bhupathiraju S.N., Rimm E.B., Spiegelman D., Chiuve S.E., Borgi L., Willett W.C., Manson J.E., Sun Q., Hu F.B. (2016). Plant-Based Dietary Patterns and Incidence of Type 2 Diabetes in US Men and Women: Results from Three Prospective Cohort Studies. PLoS Med..

[14] Oncina-Cánovas A., González-Palacios S., Notario-Barandiaran L., Torres-Collado L., Signes-Pastor A., de-Madaria E., Santibañez M., García-de La Hera M., Vioque J. (2022). Adherence to Pro-Vegetarian Food Patterns and Risk of Oesophagus, Stomach, and Pancreas Cancers: A Multi Case–Control Study (The PANESOES Study). Nutrients.

[15] Martínez C.F., Di Castelnuovo A., Costanzo S., Panzera T., Esposito S., Cerletti C., Donati M.B., de Gaetano G., Iacoviello L., Bonaccio M. (2023). Pro-Vegetarian Food Patterns and Cancer Risk among Italians from the Moli-Sani Study Cohort. Nutrients.

[16] Hargreaves S.M., Rosenfeld D.L., Moreira A.V.B., Zandonadi R.P. (2023). Plant-Based and Vegetarian Diets: An Overview and Definition of These Dietary Patterns. Eur. J. Nutr..

[17] Carey C.N., Paquette M., Sahye-Pudaruth S., Dadvar A., Dinh D., Khodabandehlou K., Liang F., Mishra E., Sidhu M., Brown R. (2023). The Environmental Sustainability of Plant-Based Dietary Patterns: A Scoping Review. J. Nutr..

[18] Willett W., Rockstrأ¶m J., Loken B., Springmann M., Lang T., Vermeulen S., Garnett T., Tilman D., DeClerck F., Wood A. (2019). Food in the Anthropocene: The EAT—Lancet Commission on Healthy Diets from Sustainable Food Systems. Lancet.

[19] Arksey H., O’Malley L. (2005). Scoping Studies: Towards a Methodological Framework. Int. J. Soc. Res. Methodol..

[20] Peters M.D., Godfrey C., McInerney P., Munn Z., Tricco A.C., Khalil H. (2020). Chapter 11: Scoping Reviews. JBI Manual for Evidence Synthesis.

[21] Page M.J., McKenzie J.E., Bossuyt P.M., Boutron I., Hoffmann T.C., Mulrow C.D., Shamseer L., Tetzlaff J.M., Akl E.A., Brennan S.E. (2021). The PRISMA 2020 Statement: An Updated Guideline for Reporting Systematic Reviews. BMJ.

[22] Radd-Vagenas S., Kouris-Blazos A., Singh M.F., Flood V.M. (2017). Evolution of Mediterranean Diets and Cuisine: Concepts and Definitions. Asia Pac. J. Clin. Nutr..

[23] Mattavelli E., Olmastroni E., Bonofiglio D., Catapano A.L., Baragetti A., Magni P. (2022). Adherence to the Mediterranean Diet: Impact of Geographical Location of the Observations. Nutrients.

[24] Ouzzani M., Hammady H., Fedorowicz Z., Elmagarmid A. (2016). Rayyan—A Web and Mobile App for Systematic Reviews. Syst. Rev..

[25] Tricco A.C., Lillie E., Zarin W., O’Brien K.K., Colquhoun H., Levac D., Moher D., Peters M.D.J., Horsley T., Weeks L. (2018). PRISMA Extension for Scoping Reviews (PRISMA-ScR): Checklist and Explanation. Ann. Intern. Med..

[26] Peters M.D.J., Marnie C., Tricco A.C., Pollock D., Munn Z., Alexander L., McInerney P., Godfrey C.M., Khalil H. (2020). Updated Methodological Guidance for the Conduct of Scoping Reviews. JBI Evid. Synth..

[27] Watling C.Z., Schmidt J.A., Dunneram Y., Tong T.Y.N., Kelly R.K., Knuppel A., Travis R.C., Key T.J., Perez-Cornago A. (2022). Risk of Cancer in Regular and Low Meat-Eaters, Fish-Eaters, and Vegetarians: A Prospective Analysis of UK Biobank Participants. BMC Med..

[28] Abd Rashid A.A., Ashari L.S., Shafiee N.H., Raja Ali R.A., Yeong Yeh L., Shahril M.R., Jan Mohamed H.J. (2023). Dietary Patterns Associated with Colorectal Cancer Risk in the Malaysian Population: A Case-Control Study with Exploratory Factor and Regression Analysis. BMC Public. Health.

[29] Nejad E.T., Moslemi E., Souni F., Mahmoodi M., Vali M., Vatanpour M., Nouri M., Ramezani A., Shateri Z., Rashidkhani B. (2023). The Association between Pro-Vegetarian Dietary Pattern and Risk of Colorectal Cancer: A Matched Case-Control Study. BMC Res. Notes.

[30] Ren X., Yu C., Peng L., Gu H., Xiao Y., Tang Y., He H., Xiang L., Wang Y., Jiang Y. (2023). Compliance with the EAT-Lancet Diet and Risk of Colorectal Cancer: A Prospective Cohort Study in 98,415 American Adults. Front. Nutr..

[31] Mohammadi F., Alijani S., Abdollahi N., Mashoufi A., Nouri M., Soltanii M., Shateri Z., Rashidkhani B. (2024). The Association between Planetary Health Diet Index and the Risk of Colorectal Cancer: A Case-Control Study. Sci. Rep..

[32] Hu F.-L., Liu J.-C., Li D.-R., Xu Y.-L., Liu B.-Q., Chen X., Zheng W.-R., Wei Y.-F., Liu F.-H., Li Y.-Z. (2025). EAT-Lancet Diet Pattern, Genetic Risk, and Risk of Colorectal Cancer: A Prospective Study from the UK Biobank. Am. J. Clin. Nutr..

[33] Yue Y., Hur J., Cao Y., Tabung F.K., Wang M., Wu K., Song M., Zhang X., Liu Y., Meyerhardt J.A. (2021). Prospective Evaluation of Dietary and Lifestyle Pattern Indices with Risk of Colorectal Cancer in a Cohort of Younger Women. Ann. Oncol..

[34] Kim J., Boushey C.J., Wilkens L.R., Haiman C.A., Le Marchand L., Park S.-Y. (2022). Plant-Based Dietary Patterns Defined by a Priori Indices and Colorectal Cancer Risk by Sex and Race/Ethnicity: The Multiethnic Cohort Study. BMC Med..

[35] Wang F., Ugai T., Haruki K., Wan Y., Akimoto N., Arima K., Zhong R., Twombly T.S., Wu K., Yin K. (2022). Healthy and Unhealthy Plant-Based Diets in Relation to the Incidence of Colorectal Cancer Overall and by Molecular Subtypes. Clin. Transl. Med..

[36] Wu B., Zhou R.-L., Ou Q.-J., Chen Y.-M., Fang Y.-J., Zhang C.-X. (2022). Association of Plant-Based Dietary Patterns with the Risk of Colorectal Cancer: A Large-Scale Case-Control Study. Food Funct..

[37] Liu F., Lv Y., Peng Y., Qiao Y., Wang P., Si C., Wang X., Gong J., Zhou H., Zhang M. (2023). Plant-Based Dietary Patterns, Genetic Predisposition and Risk of Colorectal Cancer: A Prospective Study from the UK Biobank. J. Transl. Med..

[38] Yarmand S., Rashidkhani B., Alimohammadi A., Shateri Z., Shakeri M., Sohrabi Z., Nouri M. (2024). A Healthful Plant-Based Diet Can Reduce the Risk of Developing Colorectal Cancer: Case-Control Study. J. Health Popul. Nutr..

[39] Zhong G.-C., Li Z., You A.-J., Zhu Q., Wang C.-R., Yang P.-F. (2023). Plant-Based Diets and the Risk of Pancreatic Cancer: A Large Prospective Multicenter Study. Am. J. Clin. Nutr..

[40] Di Maso M., Augustin L.S.A., Jenkins D.J.A., Crispo A., Toffolutti F., Negri E., La Vecchia C., Ferraroni M., Polesel J. (2024). Adherence to a Cholesterol-Lowering Diet and the Risk of Pancreatic Cancer: A Case—Control Study. Nutrients.

[41] Shyam S., Greenwood D.C., Mai C.-W., Tan S.S., Yusof B.-N.M., Moy F.M., Cade J.E. (2023). Major Dietary Patterns in the United Kingdom Women’s Cohort Study Showed No Evidence of Prospective Association with Pancreatic Cancer Risk. Nutr. Res..

[42] Kim J., Setiawan V.W., Wilkens L.R., Le Marchand L., Park S.-Y. (2023). Healthful Plant-Based Dietary Pattern and Risk of Hepatocellular Carcinoma in a Multiethnic Population: A Cohort Study. Am. J. Clin. Nutr..

[43] Dong X., Zhang M., Shu J., Li Y., Tan P., Peng T., Lu J., Zhang Y., Zhong X., Fang A. (2025). The Quality of Plant-Based Diets and Liver Cancer Incidence and Liver Disease Mortality in the UK Biobank. Clin. Nutr. ESPEN.

[44] Liu Y., Yang W., VoPham T., Ma Y., Simon T.G., Gao X., Chan A.T., Meyerhardt J.A., Giovannucci E.L., Zhang X. (2021). Plant-Based and Animal-Based Low-Carbohydrate Diets and Risk of Hepatocellular Carcinoma Among US Men and Women. Hepatology.

[45] Zhang X., He F., Li J., Chen R., Li X., Li L., Liu F., Wang S., Wei W. (2024). Plant-Based Dietary Patterns and Risk of Esophageal Cancer: A Prospective Cohort Study Spanning 17 Years. Chin. J. Cancer Res..

[46] Cai Y., Hong C., Han J., Fan L., Xiao X., Xiao J., Wei Y., Zhu Y., Tian J., Zhu X. (2024). Healthy Dietary Patterns, Genetic Risk, and Gastrointestinal Cancer Incident Risk: A Large-Scale Prospective Cohort Study. Am. J. Clin. Nutr..

[47] Kim J., Khil J., Kim H., Keum N.N., Zhang X., Giovannucci E. (2023). Plant-Based Dietary Patterns and the Risk of Digestive System Cancers in 3 Large Prospective Cohort Studies. Eur. J. Epidemiol..

[48] Turati F., Mignozzi S., Esposito G., Bravi F., D’Angelo A., Alicandro G., Garavello W., Augustin L.S.A., Vitale S., Giacosa A. (2025). Indices of Healthy and Unhealthy Plant-Based Diets and the Risk of Selected Digestive Cancers. Clin. Nutr..

[49] Steck S.E., Guinter M., Zheng J., Thomson C.A. (2015). Index-Based Dietary Patterns and Colorectal Cancer Risk: A Systematic Review. Adv. Nutr..

[50] Moazzen S., Cortes-Ibanez F.O., van der Vegt B., Alizadeh B.Z., de Bock G.H. (2022). Diet Quality Indices and Gastrointestinal Cancer Risk: Results from the Lifelines Study. Eur. J. Nutr..

[51] Xie L., Li Y., Song Z., Huang Y., Yao Q. (2025). Plant-Based Diet and Colorectal Cancer: A Systematic Review and Meta-Analysis of Prospective Cohort Studies. J. Gastrointest. Surg..

[52] Madrigal-Matute J., Bañón-Escandell S. (2023). Colorectal Cancer and Microbiota Modulation for Clinical Use. A Systematic Review. Nutr. Cancer.

[53] Hardt L., Mahamat-Saleh Y., Aune D., Schlesinger S. (2022). Plant-Based Diets and Cancer Prognosis: A Review of Recent Research. Curr. Nutr. Rep..

[54] Chu A.H., Lin K., Croker H., Kefyalew S., Becerra-Tomás N., Dossus L., González-Gil E.M., Ahmadi N., Park Y., Krebs J. (2025). Dietary Patterns and Colorectal Cancer Risk: Global Cancer Update Programme (CUP Global) Systematic Literature Review. Am. J. Clin. Nutr..

[55] Monteiro C.A., Cannon G., Levy R.B., Moubarac J.-C., Louzada M.L., Rauber F., Khandpur N., Cediel G., Neri D., Martinez-Steele E. (2019). Ultra-Processed Foods: What They Are and How to Identify Them. Public. Health Nutr..

[56] Wang L., Du M., Wang K., Khandpur N., Rossato S.L., Drouin-Chartier J.-P., Steele E.M., Giovannucci E., Song M., Zhang F.F. (2022). Association of Ultra-Processed Food Consumption with Colorectal Cancer Risk among Men and Women: Results from Three Prospective US Cohort Studies. BMJ.

[57] Zhong G., Zhu Q., Cai D., Hu J., Dai X., Gong J., Sun W. (2023). Ultra-processed Food Consumption and the Risk of Pancreatic Cancer in the Prostate, Lung, Colorectal and Ovarian Cancer Screening Trial. Intl J. Cancer.

[58] Zhao L., Zhang X., Yu D., Wang L., Shrubsole M.J., Zheng W., Sudenga S.L., Zhang X. (2024). Ultra-Processed Products and Risk of Liver Cancer: A Prospective Cohort Study. Clin. Nutr..

[59] Morales-Berstein F., Biessy C., Viallon V., Goncalves-Soares A., Casagrande C., Hémon B., Kliemann N., Cairat M., Blanco Lopez J., Al Nahas A. (2024). Ultra-Processed Foods, Adiposity and Risk of Head and Neck Cancer and Oesophageal Adenocarcinoma in the European Prospective Investigation into Cancer and Nutrition Study: A Mediation Analysis. Eur. J. Nutr..

[60] Meine G.C., Picon R.V., Espírito Santo P.A., Sander G.B. (2024). Ultra-Processed Food Consumption and Gastrointestinal Cancer Risk: A Systematic Review and Meta-Analysis. Am. J. Gastroenterol..

[61] Vithayathil S., Walizada M., Siddi G., Gumireddy S.R., Anto M., Elkashif I., Yu A.K. (2025). Navigating Evidence on Ultra-Processed Foods and Cancer: A Narrative Review. Cureus.

[62] Kantilafti M., Magiakou E., Chrysostomou S. (2025). Ultra-Processed Foods and Cancer Risk: A Narrative Review of Epidemiological Findings and Biological Mechanisms. Int. J. Food Sci. Nutr..

[63] Katz D.L., Meller S. (2014). Can We Say What Diet Is Best for Health?. Annu. Rev. Public. Health.

[64] Zhao Y., Zhan J., Wang Y., Wang D. (2022). The Relationship Between Plant-Based Diet and Risk of Digestive System Cancers: A Meta-Analysis Based on 3,059,009 Subjects. Front. Public. Health.

[65] Hemler E.C., Hu F.B. (2019). Plant-Based Diets for Personal, Population, and Planetary Health. Adv. Nutr..

[66] Lane M.M., Gamage E., Du S., Ashtree D.N., McGuinness A.J., Gauci S., Baker P., Lawrence M., Rebholz C.M., Srour B. (2024). Ultra-Processed Food Exposure and Adverse Health Outcomes: Umbrella Review of Epidemiological Meta-Analyses. BMJ.

[67] Chang K., Gunter M.J., Rauber F., Levy R.B., Huybrechts I., Kliemann N., Millett C., Vamos E.P. (2023). Ultra-Processed Food Consumption, Cancer Risk and Cancer Mortality: A Large-Scale Prospective Analysis within the UK Biobank. EClinicalMedicine.

[68] Jankovic N., Steppel M.T., Kampman E., de Groot L.C., Boshuizen H.C., Soedamah-Muthu S.S., Kromhout D., Feskens E.J. (2014). Stability of Dietary Patterns Assessed with Reduced Rank Regression; the Zutphen Elderly Study. Nutr. J..

[69] Newby P.K., Weismayer C., Akesson A., Tucker K.L., Wolk A. (2006). Long-Term Stability of Food Patterns Identified by Use of Factor Analysis among Swedish Women. J. Nutr..

