# Supplementary material for: Adherence to Plant-Based Dietary Patterns and Digestive Cancers: A Scoping Review
Source: Nutrients. 2026 Feb 26;18(5):756. doi: 10.3390/nu18050756 (PMC12986450; doi:10.3390/nu18050756)
Supplement: Supplementary file 1 [file nutrients-18-00756-s001.zip › nutrients-4122404-supplementary.pdf]

Table S1. Risk of bias assessment using Newcastle-Ottawa Scale adapted for case-control studies

Study-Year	Selection				Comparability	Exposure			Total Score
	Adequate definition of patient cases	Representativeness of patient cases	Selection of controls	Definition of controls	Control for important or additional factors	Ascertainment of exposure	Same method of ascertainment for cases and controls	Nonresponse rate	
Abd Rashid et al. (2023)	*	*	*	*	**	*	*	*	9
Di Maso et al. (2024)	*	*	*	*	**	*	*		8
Mohammadi et al. (2024)	*	*		*	**	*	*		7
Nejad et al. (2023)	*	*		*	**	*	*		7
Oncina-Cánovas et al. (2022)	*	*		*	**	*	*	*	8
Turati et al. (2025)	*	*	*	*	**	*	*		8
Wu et al. (2022)	*		*	*	**	*	*		7
Yarmand et al. (2024)		*	*	*	**	*	*		8

Table S2. Risk of bias assessment using Newcastle-Ottawa Scale adapted for cohort studies

Study-Year	Selection				Comparability	Exposure			Total Score
	Representativeness of the exposed cohort	Selection of the non exposed cohort	Ascertainment of exposure	Demonstration that outcome of interest was not present at start of study	Comparability of cohorts on the basis of the design or analysis	Assessment of outcome	Was follow-up long enough for outcomes to occur	Adequacy of follow up of cohorts	
Cai et al. (2024)	*	*	*	*	**	*		*	8

Dong et al. (2025)	*	*	*	*	**	*		*	8
Hu et al. (2025)	*	*	*	*	**	*		*	9
Kim et al. (2023a)	*	*	*	*	**	*	*	*	9
Kim et al. (2023b)	*	*	*	*	**	*	*	*	9
Kim et al. (2022)	*	*	*	*	**	*	*	*	9
Liu et al. (2023)	*	*	*	*	**	*	*		8
Liu et al. (2021)	*	*	*	*	**	*	*		8
Martinez et al. (2023)	*	*	*	*	**	*	*		8
Ren et al. (2023)	*	*	*	*	**	*	*		8
Shyam et al. (2023)	*	*	*	*	**	*	*		8
Wang et al. (2022)	*	*	*	*	**	*	*	*	9
Watling et al. (2022)	*	*	*	*	**	*	*		8
Yue et al. (2021)	*	*	*	*	**	*	*		8
Zhang et al. (2024)	*	*	*	*	**	*	*		8
Zhong et al. (2023)	*	*	*	*	**	*	*		8
